# Assessing the Internal and External Validity of Mobile Health Physical Activity Promotion Interventions: A Systematic Literature Review Using the RE-AIM Framework

**DOI:** 10.2196/jmir.2745

**Published:** 2013-10-04

**Authors:** Kacie CA Blackman, Jamie Zoellner, Leanna M Berrey, Ramine Alexander, Jason Fanning, Jennie L Hill, Paul A Estabrooks

**Affiliations:** ^1^Department of Human Nutrition, Foods, and ExerciseVirginia TechBlacksburg, VAUnited States; ^2^Fralin Translational Obesity Research CenterVirginia TechBlacksburg, VAUnited States; ^3^Department of Kinesiology and Community HealthUniversity of Illinois at Urbana-ChampaignUrbana, ILUnited States; ^4^Department of Family MedicineVirginia Tech Carilion Medical SchoolRoanoke, VAUnited States

**Keywords:** physical activity, mobile technology, review, generalizability

## Abstract

**Background:**

Mobile health (mHealth) interventions are effective in promoting physical activity (PA); however, the degree to which external validity indicators are reported is unclear.

**Objective:**

The purpose of this systematic review was to use the RE-AIM (reach, effectiveness, adoption, implementation, and maintenance) framework to determine the extent to which mHealth intervention research for promoting PA reports on factors that inform generalizability across settings and populations and to provide recommendations for investigators planning to conduct this type of research.

**Methods:**

Twenty articles reflecting 15 trials published between 2000 and 2012 were identified through a systematic review process (ie, queries of three online databases and reference lists of eligible articles) and met inclusion criteria (ie, implementation of mobile technologies, target physical activity, and provide original data). Two researchers coded each article using a validated RE-AIM data extraction tool (reach, efficacy/effectiveness, adoption, implementation, maintenance). Two members of the study team independently abstracted information from each article (inter-rater reliability >90%) and group meetings were used to gain consensus on discrepancies.

**Results:**

The majority of studies were randomized controlled trials (n=14). The average reporting across RE-AIM indicators varied by dimension (reach=53.3%, 2.67/5; effectiveness/efficacy=60.0%, 2.4/4; adoption=11.1%, 0.7/6; implementation=24.4%, 0.7/3; maintenance=0%, 0/3). While most studies described changes in the primary outcome (effectiveness), few addressed the representativeness of participants (reach) or settings (adoption) and few reported on issues related to maintenance and degree of implementation fidelity.

**Conclusions:**

This review suggests that more focus is needed on research designs that highlight and report on both internal and external validity indicators. Specific recommendations are provided to encourage future mHealth interventionists and investigators to report on representativeness, settings, delivery agents for planned interventions, the extent to which protocol is delivered as intended, and maintenance of effects at the individual or organizational level.

## Introduction

The numerous health benefits of physical activity (PA) are well known, but still it is estimated that roughly 31% of the world’s adult population (28% men, 34% women) is classified as insufficiently active [1]. Likewise, it is a concern in the United States where only 6-11% of children [2] and 8.2% of adults meet the national PA guidelines based on objective PA assessments [3]. Given these low PA rates, there is a need for increased attention to the development of effective and scalable PA promotion interventions that can reach a large number of people at a low cost [4].

One such approach is the use of mobile technology, since ownership is on the rise in adults and children [5,6]. By 2012, it was estimated that there were 7 billion mobile-connected devices across the globe and the number of mobile devices outnumbered the human population [7]. In the United States, according to a 2012 nationally representative survey, more than 88% of American adults own mobile phones, which is an 11% increase from 2011 [8]. Fifty-three percent of American mobile phone owners own a smartphone [8]. Furthermore, roughly 75% of 12-17 year olds own mobile phones and this is a drastic surge (ie, up 30%) from 2004 [6].

This growth in mobile technology ownership has led to the development of a number of mobile health (mHealth) intervention reviews [9-12]. Specifically, related to PA, mHealth interventions that deliver information and behavioral strategies through short message service (SMS) via mobile phones have been developed to increase PA [13-17]. In addition, ecological momentary interventions through palmtop computers and mobile phones [14] can enhance interventions and aid in improving health outcomes. The potential utility of mHealth interventions to promote PA is also evident in the large number of commercially available fitness applications that promote behavioral tracking (eg, Nike+Running, Runtastic), link to external technology devices (eg, Fitbit), or directly encourage different intensities of PA (eg, Zombies, Run!). A recent meta-analysis on the use of mobile devices [10] and text messaging review [9] for PA promotion summarized the literature in this area and concluded that interventions delivered through this modality were effective for increasing PA. Similarly, a review of Internet-based PA interventions concluded that interactive technology interventions were effective for PA promotion [18]. However, a recent Cochrane review of mHealth interventions for preventive health care suggested that the availability of studies using randomized controlled trials was insufficient to determine if these approaches could influence PA or other health behaviors [12].

Despite the popularity of commercially available health-related applications, there is little evidence that mobile phone-based interventions with demonstrated efficacy have been translated beyond the research setting and been broadly adopted [19]. Some potential reasons for the lack of translation of these interventions into more widespread use are that the scientific approach typically emphasizes high internal validity at the expense of external validity [20] and that the traditional research pace impedes the flow of disseminating relevant findings [21]. To date, reviews of mHealth interventions have evaluated the quality of studies through the lens of internal validity and emphasized improved reporting on potential confounding factors [22]. As a result, the conclusions are largely limited to factors related to intervention efficacy and the extent to which these mHealth interventions report on or achieve external validity to different settings and populations is unclear [13-17,23]. This issue was recently underscored by the publication of the CONSORT-EHEALTH reporting standards [24]. The standards included eight highly recommended and four essential categories of reporting, which highlight the need for additional attention to external validity. Briefly, the four essential categories include (1) reporting on the context within which participants accessed the intervention, (2) the delivery mode, features, and functionality of the intervention, (3) the use of prompts to interact with the intervention, and (4) any co-interventions that may occur.

To improve the reporting across behavioral interventions, Glasgow and colleagues developed the RE-AIM (reach, effectiveness, adoption, implementation, maintenance) framework to evaluate the degree to which behavioral interventions, including those targeting PA, report on internal and external validity factors [25]. The framework specifies standards related to the reporting of “Reach” into the target population and representativeness of the study sample; “Efficacy/effectiveness” of the intervention on the primary outcome tested under either optimal or real-world conditions, quality of life, and avoidance of unintended or negative consequences; “Adoption” rates of organizations and staff that would ultimately use the intervention and the characteristics of those organizations and staff; the degree to which the intervention is “Implemented” as intended; and the “Maintenance” of effects at the individual level and sustainability of the intervention at an organizational or delivery level (RE-AIM) [26]. The RE-AIM framework has demonstrated utility in summarizing reports of internal and external validity factors across numerous bodies of literature (eg, weight loss maintenance, health literacy, tobacco use, and PA interventions for older adults and for breast cancer survivors) [27-37]. Collectively, these previous reviews have provided recommendations and future directions to enhance the likelihood of research to practice. Many of these recommendations align with those proposed in the CONSORT-EHEALTH standards [24]. In particular, the context within which participants access mHealth interventions is documented within the adoption (ie, description of intervention location and staff) and each of the other three essential standards are captured within an assessment of the implementation dimension (ie, cost, intervention description including frequency, type, and duration of contacts). The primary purpose of this systematic review is to determine the degree to which studies testing mHealth interventions to promote PA report on factors that inform generalizability across settings and populations. Recommendations to improve the likelihood of broad dissemination of effective mHealth interventions are also provided based on the literature [24,27-38].

## Methods

### Selection of Studies for Review

**Figure 1:**
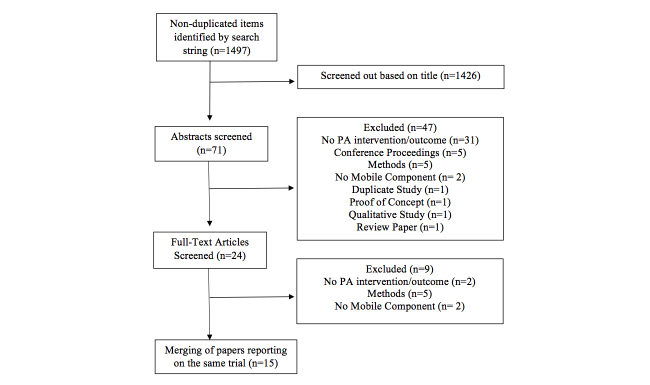
Flow diagram of study selection.

We replicated the search strategy used in a recently published meta-analysis publication that focused solely on effectiveness of mHealth interventions for PA promotion at the individual level [10]. Our literature search was conducted between August 2011 and July 2012 and included articles published between 2000 and 2012 that met the inclusion criteria indicated in Table 1. Review articles, observational (eg, cross-sectional, descriptive) commentaries, methodological articles, and articles not explicitly related to PA were excluded. Implementation of mobile technologies included data collection or conveyance of intervention information via SMS or native mobile device software or hardware. The search strategies to identify eligible articles included queries using three online databases (PsycINFO, PubMed, and Scopus) and a hand search of reference lists for articles that met inclusion criteria. The search terms included mobile phone, cell phone, PDA, SMS, or text messaging combined with PA or exercise [10]. In addition to comprehensively evaluating the reporting of RE-AIM criteria on a single trial, data was extracted from companion articles (eg, qualitative/quantitative methods measuring implementation) of studies that met inclusion criteria. Figure 1 outlines the identification of the 20 articles representing 15 trials that were included in this systematic review.

**Table 1:** Inclusion criteria.

Data type	Inclusion criteria
Participants	Any age
Language	English
Study design	Experimental and quasi-experimental
Control condition	Any comparator including active control, inactive control, or participants as their own control (ie, pre- and post-measures)
Intervention	Implementation of mobile technologies
Measurement	Assesses physical activity directly among participants
Primary outcome	Physical activity
Type of data	Original, quantitative outcome data

### Assessment of Reporting Comprehensiveness Across RE-AIM Dimensions

Comprehensiveness of reporting was determined using a previously developed 21-item validated data extraction tool that included both internal and external validity indicators based on the RE-AIM framework [27,28,31-33]. Table 2 includes details on each of the indicators assessed across the RE-AIM framework. In addition, we examined the degree to which reporting across the implementation dimension of RE-AIM addressed essential CONSORT-EHEALTH standards in terms of application costs, intervention features, theoretical backgrounds, prompts, and co-interventions [24]. Finally, due to the emerging nature of this body of literature, we also documented whether studies reported on adaptations that were made across intervention testing [34]. This was useful in order to determine the extent to which intervention fidelity was maintained during intervention implementation while allowing adaptations to the intervention to be made by delivery agents/organizations/systems to improve feasibility/acceptability/utility [39].

### Coding Protocol and Scoring

All studies were coded independently by two members of the research team with the exception of the first three studies which were coded by five members of the research team to promote familiarity with the data extraction tool. For each of the 21 items, coders indicated whether or not the indicator was reported (ie, yes or no), and subsequently extracted specific data. After independently coding, the Kappa statistic [41] was calculated to evaluate inter-rater reliability. The average Kappa statistic for consistency of coding was 0.90, indicating strong inter-rater reliability. For the differences that did arise, researchers met to discuss articles, resolve uncertainty, and gain consensus in the coding by revisiting the specific article.

To calculate the proportion reporting for each item, the number of “yes” codes was summed across the 15 studies and then divided by 15. Then the resulting number became the proportion reporting for that particular item. An overall comprehensiveness of reporting score for each article was calculated based on the number of reported indicators (possible score 0-21). Comprehensiveness of reporting score categories have been published in a past RE-AIM review [28], with articles scoring 15-21, 8-14, and less than 8, considered as high, moderate, and low quality reporting, respectively.

**Table 2:** RE-AIM internal and external validity indicators.

RE-AIM dimension	Indicator	Description	Importance
**Reach**			
	Individual level	The number, proportion, and representativeness of participants.	
	Method to identify target population	Describe the process by which the target population was identified for participation in the intervention.	Helps investigators develop an approach to determining who may be suitable for the intervention. Examples include using an electronic medical record query or mass media approaches [20].
	Inclusion criteria	Explicit statement of characteristics of the target population that were used to determine if a potential participant was eligible to participate.	Inclusion criteria should be as inclusive as possible to improve the external validity of findings [40].
	Exclusion criteria	Explicit statement of characteristics that would prevent a potential participant from being eligible to participate.	Exclusion criteria should be considered carefully to prevent potential harm to prospective participants, but should also avoid excluding individuals based on criteria that could be related to SES (eg, ability to travel to intervention site), comorbidities, or other factors that could influence an externally valid depiction of intervention effects [40].
	Participation rate	Sample size divided by the target population denominator.	Provides information on the acceptability of the study and interventions from the perspective of the target population [26].
	Representativeness	Explicit statement of characteristics of the study participants in comparison to the target population.	Identifies disparities in participation and informs the degree to which the study results are generalizable to the target population [26].
**Efficacy/effectiveness**			
	Individual level	The measure of the primary outcome, quality of life, and on avoiding unintended negative consequences.	
	Measures/results for at least 1 follow-up	The study variable(s) are measured at a time point after baseline.	To evaluate whether the intervention outcomes were statistically significant or changed (positively/negatively) [26].
	Intent-to-treat analysis utilized	Analyzing participants in trials in the groups to which they were randomized, regardless of whether they received or adhered to the allocated intervention.	Reduces bias from omitting individuals who were lost to follow-up and improves generalizability [63].
	Quality-of-life (QOL) or potential negative outcomes	QOL: Includes a measure of quality of life with some latitude for coding articles that refer to well-being or satisfaction with life. Negative outcomes: To evaluate unanticipated consequences and results that may be a product of the intervention and may have caused unintended harm.	Provide a metric to compare across interventions with different behavioral targets and provides a better sense of the impact that the intervention on the participants’ perceptions of health [26]. Allows for the weight of the harms and benefits of an intervention [26].
	Percent attrition	The proportion that was lost to follow-up or dropped out of the intervention.	High attrition lowers statistical power and treatment-correlated attrition of participants from conditions threatens internal validity [42].
**Adoption**			
	Organizational level (setting and staff)	The number, proportion, and characteristics of adopting organizations and staff.	
	Description of intervention location	The explicit statement of characteristics of the location of the intervention.	Provides an understanding of resources needed for future researchers [26].
	Description of staff who delivered intervention	The explicit statement of characteristics of the staff who delivered the intervention.	Provides information on the characteristics may be needed to deliver an intervention and assist with retention of participants [35].
	Method to identify staff who delivered intervention (target delivery agent)	Describe the process by which the staff was identified for participation in the study.	Helps investigators develop an approach to identify and engage staff that may be suitable for intervention delivery [35].
	Level of expertise of delivery agent	Training or educational background in of those delivering the intervention.	Allows for the assessment of generalizability of those delivering an intervention to typical practice settings delivery [35].
	Inclusion/exclusion criteria of delivery agent or setting	The explicit statement of characteristics of the setting/agent that were used to determine if a potential setting/agent is eligible to participate.	Inclusion criteria should be as inclusive as possible to improve the external validity of findings. Exclusion criteria should not systematically remove potential settings or staff that typical in the practice domain [20].
	Adoption rate of delivery agent or setting	The number of participating delivery settings or agents divided by the number of eligible and approached delivery settings or agents.	Provides information on the acceptability of the study and interventions from the perspective of the setting and staff that will ultimately be responsible for intervention delivery [26].
**Implementation**			
	Organizational level	The degree to which the intervention is delivered as intended.	
	Intervention duration and frequency	Duration: length the intervention over days, weeks, and months as well as the length of each intervention contact. Frequency: number of contacts with participants	Useful for replication and comparison of resources needed to resources available in a practice setting [26].
	Extent protocol delivered as intended (%)	Description of fidelity to the intervention protocol.	This provides insight into the feasibility of delivering all components of an intervention at the pre-determined date and time [26].
	Measures of cost of implementation	The ongoing cost (eg, money, time) of delivery across all levels of the intervention.	This is helpful for future researchers to be able to determine if conducting a specific intervention has economically feasible delivery [35].
**Maintenance**			
	Individual and organization level	The measure of behavior at the individual level and sustainability of the intervention at an organizational level.	
	Assessed outcomes ≥ 6 months post intervention	Description of follow-up outcome measures of individuals available at some duration after intervention termination.	Provides information on the maintenance of intervention outcomes over time [26].
	Indicators of program level maintenance	Description of program continuation after completion of the research study.	Provides information on whether the intervention can be integrated into an existing system/organization [26].
	Measures of cost of maintenance	The ongoing cost of maintaining delivery across all levels of the intervention.	Sustainability costs provides information for practice settings to determine the resources needed for long-term intervention delivery [28].

## Results

### Study Characteristics

All trials were published after 2006 and 13 were conducted in Western countries. Six studies were conducted in the United States [43-52], three trials were conducted in the United Kingdom [53-55], three trials were conducted in Australia [56-59], and one each was conducted in Hong Kong [60], New Zealand [61], and Taiwan [62]. The majority of studies were randomized controlled trials and one was a quasi-experimental trial without a control group [47-49]. Most studies intervened at the individual level, two intervened at the group level [43-45,58,59], and one targeted both levels [52]. The length of the studies ranged from 2 weeks to 2 years, with an average of 19 weeks. The most commonly reported intervention length was 12 weeks.

Five studies measured PA only through self-report [43-45,50,52,56,57], four used objective measures [46,52,55,62], and three used both self-report and objective measures [47-49,53,61]. Of the seven studies that objectively measured PA, half of the studies used a pedometer [47-49,52,60,61]. Each of the following objective PA measures were collected once: both biaxial and triaxial accelerometers [58,59], uniaxial accelerometers [53], biaxial accelerometers [51], the walking distance of the incremental shuttle walking test [62], and a mobile sensing platform [46].

In addition to PA, the majority of studies (n=11) reported on other outcomes. Eight studies reported on body mass index (BMI) [43-49,53,54,58-60,62]; two on BMI-z scores [58,59,61]; five studies reported on physiological outcomes [43-45,51,53,61,62]; four studies on psychological outcomes [47-49,53,58-60]; three studies on weight [43-45,53,54]; two each on sedentary activity/screen time [52,58,59]; diet [43-45,58,59]; and percent body fat [51,60]; and one on each on sugar sweetened beverages intake [50]; upper body muscular endurance and core abdominal isometric muscular endurance [58,59]; waist-to-hip ratio [54]; and waist circumference [43-45].

The types of mobile devices used were similar across studies. Nearly all studies (n=13) used mobile phones while two used personal digital assistants [43-45,53]. Most frequently, mobile technology was implemented as a way to monitor outcomes via self-report [43-45,51-53] or data from an external pedometer/accelerometer was manually entered on the mobile phone [50,53]. Additionally, mobile technology was used to provide prompts [47-49,54,61] to encourage behavior change [55,56] and provide health promotion information sent through SMS [58,59]. Furthermore, in two studies, mobile technology was used as an interactive mobile application [46,57], in one study to deliver an exercise program [62] and in another study as a mobile PA diary [47-49]. Table 3 shows the overall quality of RE-AIM reporting across the 21-item validated extraction tool, which is displayed as the proportion reporting.

### Reach

Reach was the second most reported dimension at 53.3% (2.67/5). Approximately half of all studies reported on four of the five items (method used to identify target population, inclusion and exclusion criteria, and participation rate). The least reported component was representativeness, with only four studies reporting [43-45,47-49,56,62]. None of the studies reported on characteristics of dropouts. All trials reported on sample size, which ranged from 17-210 participants with a median of 78. The participation rate ranged from 48-91 with a median of 51. The methods that were used to identify the target population included utilizing existing databases [43-45,54,56], regional diabetes services [61], recruitment coordinators [51], listservs [52], and an invitation letter from stakeholders [55]. In addition to English speaking, the most common inclusion criteria were PA requirement [46,50,53,54,56,58,59], weight requirement [43-45,53], or required possession/access to a mobile phone [46-49,54,56]. The majority of studies described recruitment strategies (n=11), which happened through various ways. Eleven studies focused on child participants, while four focused on adult participants [55,58,59]. Approaches for recruiting children included sending recruitment letters home [52,63], giving a presentation at school assemblies [55], teacher referrals [58,59], and using university listservs [52]. The majority of studies that enrolled adult participants were recruited mainly through local or mass media. Local mass media strategies included distributing flyers [43-45], using voicemail announcement systems [43-45], using mailing lists [43-45], posting ads on city buses [43-45], placing newspaper announcements [47-49], email [54,57], and using local mass media outlets [50,52]. Other studies’ recruitment methods comprised obtaining names and contact information from pulmonary rehabilitation coordinators [51], contacting individuals on a weight research registry via letter or email [43-45], targeting previous trial participants [43-45], and using a market research recruitment agency [46,53].

### Efficacy/Effectiveness

Efficacy/effectiveness was the most reported dimension at 60.0% (2.4/4). All studies reported on measures or results for at least one follow-up. Approximately three quarters of the studies reported on percent attrition, which ranged from 0-53%. Four studies reported on intent-to-treat analysis [51,55,58-60], six stated present-at-follow-up analyses were used [50,52,54,55,57,62], and one used present-at-follow-up and intention-to-treat analyses [43-45]. Of the two studies that reported a high attrition rate (ie, 25% or higher) [52,56], only one [56] used intent-to-treat analysis.

The majority of studies (n=12) reported whether the trial was an efficacy or effectiveness trial. Of these studies, eight were efficacy trials [47-52,55-57,62] and four were effectiveness trials [46,57-59,62]. A little over 30% of studies reported quality of life or potential negative outcomes and found that mobile PA interventions generally improved quality and did not have any significant negative outcomes [43-45,51,53,61,62]. In terms of PA outcomes for the 14 controlled trials, six studies found that the intervention group had significant differences compared to the control group, four studies had mixed results, and four had nonsignificant differences between groups. In the only quasi-experimental, single group study included in this study, the post-assessments of PA were significantly higher than pre-assessments [47-49]. Only one study assessed cost effectiveness, which indicated that cost per participant associated with a mobile phone-based exercise program was $580 and coaching was added at a low incremental cost of US$80 [51]. Moreover, there were no significant differences in PA outcomes (ie, six-minute walk distance) between these two groups (ie, mobile-coached versus mobile self-monitored) in the study [51].

### Adoption

The average proportion reporting on Adoption items was 11% (0.7/6). Level of expertise of delivery agent was the most reported adoption component (n=5). The descriptions of staff level of expertise included a nutritionist [43-45], a master’s level prepared exercise physiologist [43-45], a research assistant [56], a behavioral counselor [56], a nurse [51], and a psychologist [52]. No studies reported on method to identify staff who delivered the intervention, description of staff who delivered the intervention, inclusion/exclusion criteria of delivery agent, or adoption rate of delivery agent.

Setting-level reporting was similar to staff-level reporting. Only five studies specified the intervention location: a school [58,59,62], a research center physiologist, and an outpatient setting from four regional adolescent diabetes services [61]. Last, only two studies described the intervention location [58,59,62], two studies noted inclusion/exclusion criteria of setting [58,59,62], and one indicated adoption rate of setting [58,59].

### Implementation

The average proportion reporting on Implementation indicators was 24% (0.7/3). Intervention duration and frequency were the most frequently reported items (n=6) [43-45,50-52,57-59]. Few studies reported on measures of cost of implementation (n=3) [51,58,59,62] or the degree to which the intervention protocol was carried out as intended (n=2) [53,58,59]. Though it appeared that no study charged participants for the applications, none explicitly stated this. Further, no study reported on adaptations made to intervention during the study. More than half (n=8) of the studies had a theoretical basis [43-46,56,58,59,62], with social cognitive theory used most frequently (n=3) [50,56,58,59]. Almost all studies (n=13) stated the degree to which participants received prompts, co-interventions, and other intervention components, including methods such as self-monitoring of outcomes through mobile technology (eg, mobile phone or PDA), class attendance, application usage, or the completion of intervention.

### Maintenance

Maintenance was the dimension that was reported least among the RE-AIM dimensions, with no items (0%, 0/3) reported. The reporting on indicators of individual-level or program-level maintenance were not reported in any trial. 

### Comprehensiveness of Reporting on RE-AIM Criteria

The average comprehensiveness of reporting score was 6.9 out of a possible 21-item reporting coding sheet and scores ranged from 3-13. None of the studies were categorized as high reporting quality, six studies were moderate (range 8-11) [43-45,51,52,56,58,59,62], and nine studies were of low reporting quality [46-50,53-55,57,60,61].

**Table 3:** Proportion of mobile health interventions reporting RE-AIM dimensions and components (n=15).

RE-AIM Dimensions	RE-AIM Components	Proportion Reporting^a^, %
**Reach**		
	Method to identify target population	60.0
	Inclusion criteria	80.0
	Exclusion criteria	60.0
	Participation rate	46.7
	Representativeness	26.7
	Average across Reach Components	53.3
**Efficacy/effectiveness**		
	Measures/results for at least one follow-up	100.0
	Intent to treat analysis utilized	33.3
	Quality-of-life or potential negative outcomes	33.3
	Percent attrition	73.3
	Average across Efficacy/Effectiveness Components	60.0
**Adoption**		
	Description of intervention location	13.0
	Description of staff who delivered intervention	0.0
	Method to identify staff who delivered intervention (target delivery agent)	0.0
	Level of expertise of delivery agent	33.3
	Inclusion/exclusion criteria of delivery agent or setting	13.3
	Adoption rate of delivery agent or setting	6.7
	Average across Adoption Components	11.1
**Implementation**		
	Intervention duration and frequency	40.0
	Extent protocol delivered as intended (%)	13.3
	Measures of cost of implementation	20.0
	Average across Implementation Components	24.4
**Maintenance**		
	Assessed outcomes ≥ 6 months post intervention	0.0
	Indicators of program level maintenance	0.0
	Measures of cost of maintenance	0.0
	Average across Maintenance Components	0.0

^a^Based on denominator of 15 intervention trials, reported across 20 articles.

## Discussion

### Principal Findings

Our review highlighted a recent increase in studies conducted to determine the efficacy or effectiveness of mHealth interventions for the promotion of PA. We identified gaps across and within each of the RE-AIM dimensions, potentially as a result of the relative early stages of this area of research. We also understand that there is a need to advance research by utilizing innovative, flexible, and rapid research designs and “rapid-learning research systems” where researchers, funders, health systems, practitioners, and community partners collaborate [21]; however, the lack of internal and external validity reporting identified indicated that few innovative designs are currently being used in this area of investigation.

Still, the comprehensiveness of reporting on RE-AIM criteria across these mHealth articles was relatively low with a number of gaps in reporting on both internal (eg, extent that the protocol was delivered as intended) and external validity factors (eg, description of intervention location and staff). At the individual level (ie, reach, efficacy/effectiveness, and maintenance), the reporting on issues related to reach and maintenance are particularly problematic. At the organizational or delivery level (ie, adoption, implementation, maintenance), there are large gaps in reporting across each of the dimensions. These gaps extend to the reporting across the four CONSORT-EHEALTH standards of access as well as the degree to which intervention features and functionality were addressed. Based on our findings, the results reported on mHealth PA interventions, from both an internal and external validity perspective, should be considered with caution.

Consistent with past research, this body of literature does not typically describe the target population or give indications as to the degree to which the study samples are representative of a larger population [27,28,31,33,35,37,40,42]. Thus, inferences cannot be made regarding who may be likely to benefit from these interventions based on different demographic, economic, or behavioral factors. Similarly, it is unclear which subgroups of the population may be more or less likely to engage in mHealth PA interventions. This is especially important to document given that those studies that did examine the representativeness of the study sample to the target population found that nonparticipants were less educated [43-45,56] and, if they did engage in the study, had greater difficulty in operating technology [47-49,62]. Additionally, almost all of the studies used convenience sampling procedures rather than recruiting from a known target population denominator. It is vital to recruit larger numbers of subgroups of the target population so that individuals that could most benefit from the intervention are actually receiving it. This information, across studies, is critical to ensure that interventions are designed to address broad access to the intervention and the needs of subgroups of a target population that suffer from health disparities (eg, lower education levels).

Similar to other areas of research, efficacy or effectiveness based upon changes to the PA and percent attrition were reported consistently across the majority of studies while the maintenance of those changes were not [27-29,31,32,36,37]. However, the generally positive effects found across studies may be overestimated based on the degree to which attrition was considered in follow-up analyses. That is, only one in every three studies reported using intent-to-treat analyses, with the majority limiting the description of study results to those who were present for follow-up assessments. Given the recidivism related to physical inactivity, it may be surprising that no study examined the maintenance of PA change at least 6 months past completion of the intervention. On one hand, the area is relatively new and it appears that researchers have emphasized determining the degree to which these interventions can initiate change. On the other hand, mHealth interventions may be less likely to encourage PA change maintenance due to advancements in newer technology that could make current interventions obsolete, the potential of technical problems that may reduce motivation, or simply decreased participant engagement over time. Until maintenance is documented in mHealth PA intervention studies, it is left to researcher and participant speculations on how well these interventions can contribute to maintained PA change over a long period of time.

Organizational or delivery level facets of RE-AIM have consistently been underreported across behavior change intervention studies; yet, studies on mHealth PA interventions appear to be even less likely to report on organizational adoption, implementation, and maintenance [29,30,37-39]. To be balanced, the majority of the studies included were reported as efficacy trials and some adoption information like inclusion and exclusion criteria of the staff and locations of intervention delivery may not be relevant. Similarly, efficacy and effectiveness trials do not typically have a goal to achieve and track maintained delivery of an intervention beyond the life of a grant. However, to allow for replication and determination of generalizability, even within highly controlled efficacy trials, it is necessary to provide the description of (1) the intervention costs and location, (2) the characteristics of the intervention and those who delivered it, (3) the degree to which the intervention was delivered as intended, and (4) if any adaptations were made to the intervention during the study period. An additional potential critique of this literature is the tendency for participants to use non-assigned treatments [22] that may contribute to the intervention’s effectiveness. However, this body of literature included reports of co-interventions that, to some degree, address this issue. Still, no articles reported explicit tracking of non-assigned treatments so that possible contributions to effectiveness could be determined [22].

Understanding costs across RE-AIM dimensions is also key for dissemination [38]. In addition to implementation costs, other costs may be accrued both by organizations implementing these interventions as well as by the participants using them. For example, tracking of costs related to recruitment, equipment, technology (eg, batteries/chargers, mobile phone, service plan), and maintenance (eg, continue program once study period or funding is over) can improve the information necessary for decision making. From the perspective of the CONSORT-EHEALTH standards, future costs to the consumer should be considered in relation to the expected reach and effectiveness of mHealth PA interventions [24]. Without information on adoption and implementation, it is difficult to know the resources needed to successfully implement mHealth interventions in diverse locations or with staff of different levels of expertise.

Based upon the growth of research in the area of mHealth PA interventions and the review of this literature to date, there are a number of ways to improve the assessment and reporting on individual and organizational level factors that will improve our understanding of both the internal and external validity of this work. In Table 4, we provide a number of recommendations across RE-AIM dimensions specific to mHealth PA intervention research. In addition to these recommendations, the use of mixed method approaches that blend qualitative and quantitative data collected from participants and from those who implement the intervention could add depth to the data collected in mHealth PA studies and improve subsequent replication and implementation efforts [34]. Further, from a translational science perspective, tracking mHealth intervention costs across RE-AIM dimensions can inform adoption and delivery decisions within community and clinical practice settings.

**Table 4:** Recommendations.

RE-AIM component	Recommendations for reporting on future mHealth PA studies
**Reach**	
	Report on characteristics (eg, demographics, behavioral outcomes) of nonparticipants and compare them to participants to understand the representativeness of the study sample. If not possible for Institutional Review Board reasons to compare nonparticipants directly, participants can be compared to the general local population.
	Indicate exclusion criteria so that it is clear as to why certain individuals were not eligible for participation.
	Report on inclusion criteria (eg, computer/Internet literacy [24]) so that investigators can understand why specific individuals were selected.
	Describe recruitment methods and adaptations to recruitment methods so that future researchers will know the best ways to recruit for mHealth PA interventions.
	Recruit participants from a known denominator that are representative of the target population.
	Calculate the participation rate based upon a known denominator: # eligible approached and agreed to participate/total # eligible and approached.
	Describe how participants accessed the application, and cost to access application [24].
**Effectiveness**	
	Use intention-to-treat methods.
	Assess potential negative outcomes of the intervention and quality of life before and after the intervention.
	Indicate subgroup effects, especially those related to health equity issues.
**Adoption**	
	Report on characteristics of the location where the intervention is delivered and the staff who deliver the intervention and describe reasons for selection of this location and staff.
	If applicable, explicitly state inclusion/exclusion criteria of participating staff.
	If delivery locations or staff volunteer or are recruited for the study, calculate participation rate of settings/staff based on the number who volunteer divided by the number who were invited.
	Describe the level of human involvement required for the trial compared to the level of human involvement for a routine application [24].
	Describe the level of prompts/reminders required for the trial compared to the level of prompts/reminders for a routine application [24].
	Describe any interventions (including training sessions/support) that are implemented in addition to the targeted mHealth intervention [24].
**Implementation**	
	Report on intervention content, duration, and frequency of in-person and virtual sessions (eg, SMS, applications).
	Provide information intervention costs (eg, price of mobile technology, mobile phone data plan, time it takes to implement each session).
	Indicate percent delivered as intended (eg, text messages sent/unsent/received/not received; any application functioning problems or other technology problems).
	Reports of engagement should use standard or harmonized reporting methods (eg, number of sessions, number of bug fixes).
	Describe adaptations made to the intervention during implementation.
**Maintenance**	
	Include an assessment of maintenance of PA change 6 months after the completion of the intervention.
	Provide a description of how the intervention could be sustained or, if applicable, provide data on the degree to which the intervention is sustained over time.
	Report on strategies included during intervention design related to technical staff and potential participants to produce interventions that are functional and persuasive for a long period of time.

### Limitations

Our review includes some limitations. First, our conclusions and recommendations are based on the degree to which these studies reported on specific RE-AIM dimensions. It is possible that some of these data have been collected, but not reported. To address this, we included all available articles on any given trial. Still, investigator plans and data for maintenance/sustainability or designing for dissemination may exist but go unreported; however, a transparent reporting of any existing plans would provide additional important context for any intervention study. In addition, a lack of reporting on an outcome cannot be equated to a lack of an intervention’s ability to achieve that outcome (eg, lack of reporting on maintenance cannot be equated to a lack of maintenance). Second, because mHealth PA interventions are relatively novel and this is an emergent research area, the goal of the studies included within this review may have been to establish internal validity (eg, effectiveness of study outcomes), and therefore we must be cautious of being overly critical of these studies relative to their reporting of organizational adoption or maintenance factors.

### Conclusions

There is an emergent body of literature reporting on mHealth PA interventions. On average, the studies provide initial evidence that these interventions may have promise in helping participants initiate PA. However, few studies report on key internal (eg, delivery as intended) or external (eg, descriptions of participants, settings, and delivery staff) factors. As a result, the degree to which these findings are robust and generalizable cannot be determined. Improved reporting across RE-AIM dimensions and the use of intention-to-treat, tracking of costs, and mixed methods approaches are recommended to ensure mHealth PA interventions are developed that can be broadly applicable across target populations, intervention delivery locations, and staff of differing levels of expertise.

## Abbreviations

CONSORT-EHEALTH: Consolidated Standards of Reporting Trials of Electronic and Mobile Health Applications and online TeleHealth
mHealth: mobile health
PA: physical activity
RE-AIM: Reach, Effectiveness, Adoption, Implementation, Maintenance framework

## References

[1] (2008). World Health Organization.

[2] Trojano RP, Berrigan D, Dodd KW, Mâsse LC, Tilert T, McDowell M (2008). Physical activity in the United States measured by accelerometer. Med Sci Sports Exerc.

[3] Tucker JM, Welk GJ, Beyler NK (2011). Physical activity in U.S.: adults compliance with the Physical Activity Guidelines for Americans. Am J Prev Med.

[4] Davies CA, Spence JC, Vandelanotte C, Caperchione CM, Mummery WK (2012). Meta-analysis of internet-delivered interventions to increase physical activity levels. Int J Behav Nutr Phys Act.

[5] Smith A (2010). Pew Internet & American Life Project.

[6] Lenhart A, Ling R, Campbell S, Purcell K (2010). Pew Internet & American Life Project.

[7] (2012). Cisco Systems Inc.

[8] Smith A (2012). Pew Internet & American Life Project.

[9] Buchholz SW, Wilbur J, Ingram D, Fogg L (2013). Physical activity text messaging interventions in adults: a systematic review. Worldviews Evid Based Nurs.

[10] Fanning J, Mullen SP, McAuley E (2012). Increasing physical activity with mobile devices: a meta-analysis. J Med Internet Res.

[11] Martínez-Pérez B, de la Torre-Díez I, López-Coronado M (2013). Mobile health applications for the most prevalent conditions by the World Health Organization: review and analysis. J Med Internet Res.

[12] Vodopivec-Jamsek V, de Jongh T, Gurol-Urganci I, Atun R, Car J (2012). Mobile phone messaging for preventive health care. Cochrane Database Syst Rev.

[13] Fjeldsoe BS, Marshall AL, Miller YD (2009). Behavior change interventions delivered by mobile telephone short-message service. Am J Prev Med.

[14] Heron KE, Smyth JM (2010). Ecological momentary interventions: incorporating mobile technology into psychosocial and health behaviour treatments. Br J Health Psychol.

[15] Krishna S, Boren SA, Balas EA (2009). Healthcare via cell phones: a systematic review. Telemed J E Health.

[16] Lau PWC, Lau EY, Wong DP, Ransdell L (2011). A systematic review of information and communication technology-based interventions for promoting physical activity behavior change in children and adolescents. J Med Internet Res.

[17] Militello LK, Kelly SA, Melnyk BM (2012). Systematic review of text-messaging interventions to promote healthy behaviors in pediatric and adolescent populations: implications for clinical practice and research. Worldviews Evid Based Nurs.

[18] van den Berg MH, Schoones JW, Vliet Vlieland TP (2007). Internet-based physical activity interventions: a systematic review of the literature. J Med Internet Res.

[19] (2010). Dolan B.

[20] Estabrooks PE, Gyurcsik NC (2003). Evaluating the impact of behavioral interventions that target physical activity: issues of generalizability and public health. Psychology of Sport and Exercise.

[21] Riley WT, Glasgow RE, Etheredge L, Abernethy AP (2013). Rapid, responsive, relevant (R3) research: a call for a rapid learning health research enterprise. Clin Transl Med.

[22] Danaher BG, Lichtenstein E, McKay HG, Seeley JR (2009). Use of non-assigned smoking cessation programs among participants of a Web-based randomized controlled trial. J Med Internet Res.

[23] Fischer S, Stewart TE, Mehta S, Wax R, Lapinsky SE (2003). Handheld computing in medicine. J Am Med Inform Assoc.

[24] Eysenbach G, CONSORT-EHEALTH Group (2011). CONSORT-EHEALTH: improving and standardizing evaluation reports of Web-based and mobile health interventions. J Med Internet Res.

[25] Glasgow RE, Klesges LM, Dzewaltowski DA, Bull SS, Estabrooks P (2004). The future of health behavior change research: what is needed to improve translation of research into health promotion practice?. Ann Behav Med.

[26] Glasgow RE, Vogt TM, Boles SM (1999). Evaluating the public health impact of health promotion interventions: the RE-AIM framework. Am J Public Health.

[27] Akers JD, Estabrooks PA, Davy BM (2010). Translational research: bridging the gap between long-term weight loss maintenance research and practice. J Am Diet Assoc.

[28] Allen K, Zoellner J, Motley M, Estabrooks PA (2011). Understanding the internal and external validity of health literacy interventions: a systematic literature review using the RE-AIM framework. J Health Commun.

[29] Antikainen I, Ellis R (2011). A RE-AIM evaluation of theory-based physical activity interventions. J Sport Exerc Psychol.

[30] Bull SS, Gillette C, Glasgow RE, Estabrooks P (2003). Work site health promotion research: to what extent can we generalize the results and what is needed to translate research to practice?. Health Educ Behav.

[31] Dzewaltowski DA, Estabrooks PA, Klesges LM, Bull S, Glasgow RE (2004). Behavior change intervention research in community settings: how generalizable are the results?. Health Promot Int.

[32] Estabrooks P, Dzewaltowski DA, Glasgow RE, Klesges LM (2003). Reporting of validity from school health promotion studies published in 12 leading journals, 1996-2000. J Sch Health.

[33] Glasgow RE, Bull SS, Gillette C, Klesges LM, Dzewaltowski DA (2002). Behavior change intervention research in healthcare settings: a review of recent reports with emphasis on external validity. Am J Prev Med.

[34] Kessler RS, Purcell EP, Glasgow RE, Klesges LM, Benkeser RM, Peek CJ (2013). What does it mean to "employ" the RE-AIM model?. Eval Health Prof.

[35] Klesges LM, Dzewaltowski DA, Glasgow RE (2008). Review of external validity reporting in childhood obesity prevention research. Am J Prev Med.

[36] McMahon S, Fleury J (2012). External validity of physical activity interventions for community-dwelling older adults with fall risk: a quantitative systematic literature review. J Adv Nurs.

[37] White SM, McAuley E, Estabrooks PA, Courneya KS (2009). Translating physical activity interventions for breast cancer survivors into practice: an evaluation of randomized controlled trials. Ann Behav Med.

[38] Estabrooks PA, Allen KC (2013). Updating, employing, and adapting: a commentary on What does it mean to "employ" the RE-AIM model. Eval Health Prof.

[39] Aarons GA, Green AE, Palinkas LA, Self-Brown S, Whitaker DJ, Lutzker JR, Silovsky JF, Hecht DB, Chaffin MJ (2012). Dynamic adaptation process to implement an evidence-based child maltreatment intervention. Implement Sci.

[40] (2012). National Institutes of Health.

[41] Cohen J (1960). A Coefficient of Agreement for Nominal Scales. Educational and Psychological Measurement.

[42] Shadish WR, Cook TD, Campbell DT (2002). Experimental and quasi-experimental designs for generalized causal inference.

[43] Burke LE, Conroy MB, Sereika SM, Elci OU, Styn MA, Acharya SD, Sevick MA, Ewing LJ, Glanz K (2011). The effect of electronic self-monitoring on weight loss and dietary intake: a randomized behavioral weight loss trial. Obesity (Silver Spring).

[44] Burke LE, Styn MA, Glanz K, Ewing LJ, Elci OU, Conroy MB, Sereika SM, Acharya SD, Music E, Keating AL, Sevick MA (2009). SMART trial: A randomized clinical trial of self-monitoring in behavioral weight management-design and baseline findings. Contemp Clin Trials.

[45] Conroy MB, Yang K, Elci OU, Gabriel KP, Styn MA, Wang J, Kriska AM, Sereika SM, Burke LE (2011). Physical activity self-monitoring and weight loss: 6-month results of the SMART trial. Med Sci Sports Exerc.

[46] Consolvo S, Klasnja P, McDonald DW, Avrahami D, Froehlich J, LeGrand L, Libby R, Mosher K, Landay JA (2008). Flowers or a robot army? Encouraging awareness & activity with personal, mobile displays.

[47] Fukuoka Y, Lindgren T, Jong S (2012). Qualitative exploration of the acceptability of a mobile phone and pedometer-based physical activity program in a diverse sample of sedentary women. Public Health Nurs.

[48] Fukuoka Y, Kamitani E, Dracup K, Jong SS (2011). New insights into compliance with a mobile phone diary and pedometer use in sedentary women. J Phys Act Health.

[49] Fukuoka Y, Vittinghoff E, Jong SS, Haskell W (2010). Innovation to motivation--pilot study of a mobile phone intervention to increase physical activity among sedentary women. Prev Med.

[50] King AC, Ahn DK, Oliveira BM, Atienza AA, Castro CM, Gardner CD (2008). Promoting physical activity through hand-held computer technology. Am J Prev Med.

[51] Nguyen HQ, Gill DP, Wolpin S, Steele BG, Benditt JO (2009). Pilot study of a cell phone-based exercise persistence intervention post-rehabilitation for COPD. Int J Chron Obstruct Pulmon Dis.

[52] Shapiro JR, Bauer S, Hamer RM, Kordy H, Ward D, Bulik CM (2008). Use of text messaging for monitoring sugar-sweetened beverages, physical activity, and screen time in children: a pilot study. J Nutr Educ Behav.

[53] Hurling R, Catt M, Boni MD, Fairley BW, Hurst T, Murray P, Richardson A, Sodhi JS (2007). Using internet and mobile phone technology to deliver an automated physical activity program: randomized controlled trial. J Med Internet Res.

[54] Prestwich A, Perugini M, Hurling R (2010). Can implementation intentions and text messages promote brisk walking? A randomized trial. Health Psychol.

[55] Sirriyeh R, Lawton R, Ward J (2010). Physical activity and adolescents: an exploratory randomized controlled trial investigating the influence of affective and instrumental text messages. Br J Health Psychol.

[56] Fjeldsoe BS, Miller YD, Marshall AL (2010). MobileMums: a randomized controlled trial of an SMS-based physical activity intervention. Ann Behav Med.

[57] Kirwan M, Duncan MJ, Vandelanotte C, Mummery WK (2012). Using smartphone technology to monitor physical activity in the 10,000 Steps program: a matched case-control trial. J Med Internet Res.

[58] Lubans DR, Morgan PJ, Okely AD, Dewar D, Collins CE, Batterham M, Callister R, Plotnikoff RC (2012). Preventing Obesity Among Adolescent Girls: One-Year Outcomes of the Nutrition and Enjoyable Activity for Teen Girls (NEAT Girls) Cluster Randomized Controlled Trial. Arch Pediatr Adolesc Med.

[59] Lubans DR, Morgan PJ, Dewar D, Collins CE, Plotnikoff RC, Okely AD, Batterham MJ, Finn T, Callister R (2010). The Nutrition and Enjoyable Activity for Teen Girls (NEAT girls) randomized controlled trial for adolescent girls from disadvantaged secondary schools: rationale, study protocol, and baseline results. BMC Public Health.

[60] Cheung PPY, Chow BC, Parfitt G (2008). Using environmental stimuli in physical activity intervention for school teachers: A pilot study. Int Electron J Health Educ.

[61] Newton KH, Wiltshire EJ, Elley CR (2009). Pedometers and text messaging to increase physical activity: randomized controlled trial of adolescents with type 1 diabetes. Diabetes Care.

[62] Liu WT, Wang CH, Lin HC, Lin SM, Lee KY, Lo YL, Hung SH, Chang YM, Chung KF, Kuo HP (2008). Efficacy of a cell phone-based exercise programme for COPD. Eur Respir J.

[63] (2012). The Cochrane Collaboration.

